# Cerebrovascular Complications After Upper Extremity Access for Complex Aortic Interventions: A Systematic Review and Meta-Analysis

**DOI:** 10.1007/s00270-019-02330-6

**Published:** 2019-10-07

**Authors:** Max M. Meertens, Charlotte C. Lemmens, Gustavo S. Oderich, Geert W. H. Schurink, Barend M. E. Mees

**Affiliations:** 1 Department of Vascular Surgery, Maastricht University Medical Center, P. Debyelaan 25, 6229 HX Maastricht, The Netherlands; 2 Advanced Endovascular Aortic Research Program, Division of Vascular and Endovascular Surgery, Mayo Clinic, Rochester, MN USA; 3 European Vascular Center Aachen-Maastricht, Aachen, Germany; 4 European Vascular Center Aachen-Maastricht, Maastricht, The Netherlands

**Keywords:** Upper extremity access, Cerebrovascular events, Complex EVAR, Access complications, Systematic Review, Meta-analysis

## Abstract

**Purpose:**

The purpose of this study was to review the risk of developing cerebrovascular complications from upper extremity access during endovascular treatment of complex aortic aneurysms.

**Methods:**

A systematic review and meta-analysis were conducted according to the PRISMA guideline. An electronic search of the public domains Medline (PubMed), Embase (Ovid), Web of Science and Cochrane Library was performed to identify studies related to the treatment of aortic aneurysms involving upper extremity access. Meta-analysis was used to compare the rate of cerebrovascular event after left, right and bilateral upper extremity access. Results are presented as relative risk (RR) and 95% confidence intervals (CIs).

**Results:**

Thirteen studies including 1276 patients with complex endovascular treatment of aortic aneurysms using upper extremity access were included in the systematic review. Left upper extremity access (UEA) was used in 1028 procedures, right access in 148 and bilateral access in 100 procedures. The rate of cerebrovascular complications for patients treated through left UEA was 1.7%, through right UEA 4% and through bilateral UEA 5%. In the meta-analysis, we included seven studies involving 645 patients treated with a left upper extremity access, 87 patients through a right and 100 patients through a bilateral upper extremity access. Patients, who underwent right-sided (RR 5.01, 95% CI 1.51–16.58, *P* = 0.008) or bilateral UEA (RR 4.57, 95% CI 1.23–17.04, *P* = 0.02), had a significantly increased risk of cerebrovascular events compared to those who had a left-sided approach.

**Conclusion:**

Left upper extremity access is associated with a significantly lower rate of cerebrovascular complications as compared to right or bilateral upper extremity access.

## Introduction

The development of several complex endovascular aneurysm repair techniques has expanded the indications of endovascular aortic repair (EVAR) in more advanced aortic aneurysms, involving aneurysms with side branches or short-neck aneurysms [1–3]. The choice of treatment technique depends on aneurysm extent, urgency, physician preference and availability of stent grafts at individual centers [4]. To cannulate aortic side branches, an upper extremity access is often required as adjunctive to femoral access [5]. Consequently, these procedures are more challenging and time-consuming and can have more complications than during conventional EVARs [6–8]. For example, complex EVAR interventions are known to increase peri- and post-interventional cerebrovascular event [cerebrovascular accident (CVA), transient ischemic attack (TIA)] risks compared to conventional EVARs [2, 6]. There are different hypotheses for the cause of the increased incidence, such as arch manipulation, causing disruption of vulnerable plugs passing to the cerebral arteries, or the reduced flow in and distal to the brachiocephalic trunk due to temporary occlusion by the inserted sheath [9, 10]. Several authors make different and contradictory statements about the influence of the choice of upper extremity access site on the cerebrovascular event risk, in particular whether right upper extremity access increases the risk [10–12].

The purpose of this study was to systematically analyze and compare the cerebrovascular event risk of left, right and bilateral upper extremity access during complex endovascular aortic interventions for aortic aneurysms.

## Methods

This systematic review was registered at PROSPERO (Registration number: CRD42018108975).

### Literature Search and Study Selection

This systematic review and meta-analysis assembled clinical evidence using a prespecified protocol and an explicit, reproducible plan for literature research and synthesis as recommended by the Preferred Reporting Items of Systematic Reviews and Meta-Analyses (PRISMA) guidelines [13]. The Medline (Pubmed), Embase (Ovid), Web of Science and Cochrane Library databases were searched by two authors (MMM and CCL) in September 2018. The following search terms and medical subject headings (MESH) were used: stroke, cerebrovascular accident, “Stroke” [Mesh], MACCE, major adverse cardiac and cerebrovascular events, covered endovascular reconstruction of the aortic bifurcation, Nellix endovascular aortic repair, snorkel endovascular aortic repair, chimney endovascular aortic repair, chimney endovascular aortic sealing, branched–fenestrated endovascular aortic repair, branched endovascular aortic repair, fenestrated endovascular aortic repair and branched–fenestrated endovascular aortic repair. The database search was supplemented by scanning the references of the included studies. The retrieved records were screened on the title, abstract and result tables by both researchers, and eligibility assessment following the in- and exclusion criteria described below was performed.

### Selection Criteria

The study selection was performed by two reviewers (MMM and CCL). In case the reviewers had any disagreement, this was resolved by consensus with the senior author (BM). Studies were included in the systematic review if they met the following criteria, irrespective of the epidemiological design: (1) Full text had to be accessible via the electronic library of the University of ____Maastricht_; (2) studies including five or more human patients; (3) studies involving patients who underwent complex aortic interventions for aortic aneurysms and required an upper extremity access; (4) studies which clearly outlined the upper extremity access and peri- and postoperative cerebrovascular event rate. We excluded studies which did not differentiate the cerebrovascular event rate between the right and left upper extremity access. Furthermore, we excluded aortic arch procedures and aortic dissection treatments due to their increased cerebrovascular event risk to obtain certain homogeneity.

We included in the meta-analysis all studies that reported upper extremity laterality (right, left or bilateral) and specified outcomes for each upper extremity approach.

### Data Collection and Quality Assessment

For each included study, eligible data were retrieved by one researcher and verified by the second. The following data were extracted from each included study: first author, year, type of publication, size of study population, mean age, amount of male patients, prevalence of hypertension, preprocedural thrombocyte aggregation inhibition, aneurysm type, aneurysm diameter, intraprocedural anticoagulation, performed complex endovascular aortic intervention, upper extremity access vessel, percutaneous approach or surgical exposure of the access vessel, left upper extremity access, right upper extremity access, bilateral upper extremity access, maximal induced sheath size, wire length, procedure time, amount of included aortic side branches, procedural success, cerebrovascular event rate (CVA and TIA), cerebrovascular event severity and recovery, 30-day mortality rate and access complications. The methodological quality of all included studies was assessed using the checklist recommended by the 9-point Newcastle–Ottawa scale (NOS) [14].

### Statistical Analysis

Meta-analyses were used to compare studies that treated patients both through left upper extremity approach and through either right or bilateral upper extremity approach for complex endovascular aortic repair. Random effects meta-analyses were performed using the Mantel–Haenszel method for dichotomous data to estimate the pooled relative risk (RR). Statistical heterogeneity was assessed using *I*^2^ statistics. Studies with an *I*^2^ > 50% were considered to have significant heterogeneity. *P* values of < 0.05 were considered as statistical significance. The meta-analysis was run in RevMan (version 5.3). Hazard plots were generated using Excel 2010 for Windows 7.

## Results

### Study Selection

**Fig. 1:**
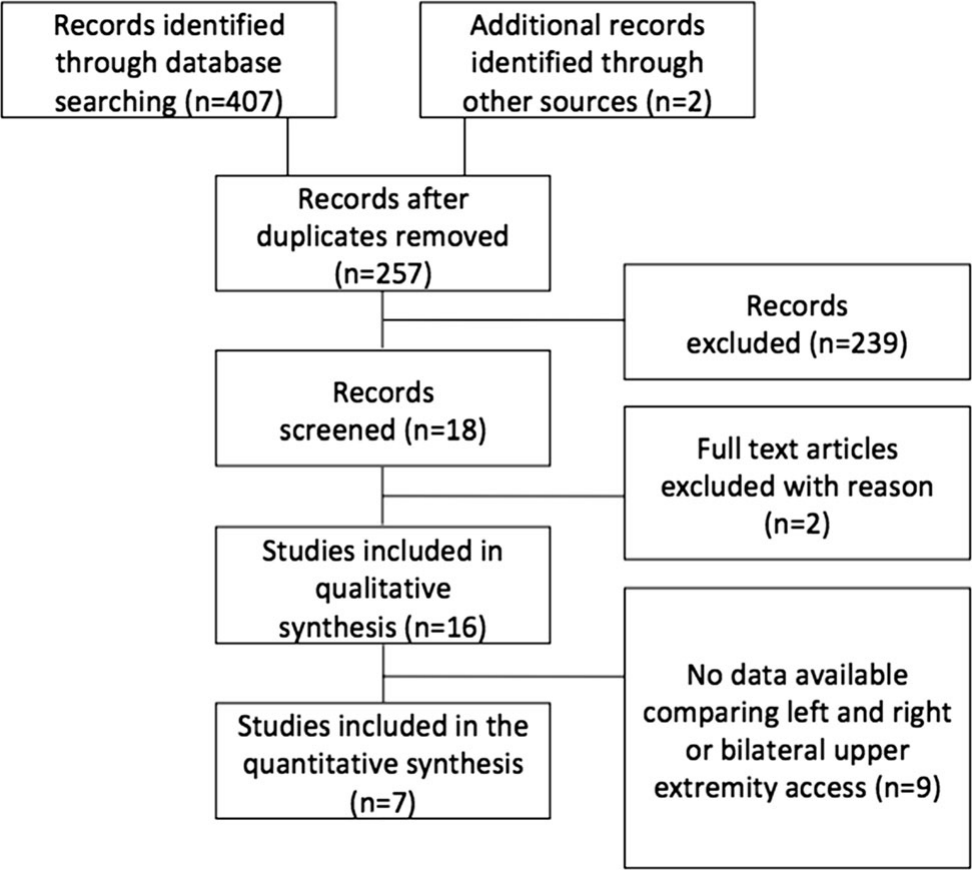
Flow diagram of study selection

Our search identified 407 articles (Fig. 1) and was condensed to 257 eligible studies after the removal of duplicates. Two articles were added from the manual search of references [15, 16]. After screening the records, 239 articles were removed, because they focused on aortic arch interventions or aortic dissection, or did not have cerebrovascular events as an outcome or did not report the side of upper extremity access. No articles were excluded for language issues. All identified publications were cohort studies or case series. No randomized controlled trials were identified in the existing literature. We excluded two [15, 17] further studies. Both studies did present the overall stroke rate as well as the number of patients accessed from each upper limb, but it was not possible to determine how many strokes occurred after which access strategy. The final systematic review included 16 studies (11 retrospective cohort studies and five case series) [10–12, 16, 18–29] of which three were congress abstracts [11, 22, 28].

### Patient Demographics and Study Characteristics

The mean patient age was 74.4 and ranged from 70 to 77 years. The majority of patients were male in all studies, ranging from 66 to 95%. The prevalence of hypertension varied between 69 and 100%. One study presented neither age and gender nor prevalence of hypertension (Table 1) [22]. Four studies did report on the number of patients on preprocedural thrombocyte aggregation inhibition, ranging from 66 to 100% [10, 12, 20, 27]. Aneurysm extent included 262 thoracoabdominal aortic aneurysms [10, 11, 21, 27–29] and 867 complex (from juxtarenal to paravisceral) abdominal aortic aneurysms [10–12, 16, 18–20, 23–25, 27, 28]. Two studies did not provide aneurysm classification [22, 26]. The mean diameter was reported by seven studies and ranged from 50 mm to 74 mm, and the overall mean diameter was 64 mm [11, 18, 19, 23–25]. Forty-five patients were treated in an acute setting (Table 1) [10, 12]. All studies were hospital-based and embraced vascular surgery patients. Seven studies were from Europe, six from the USA, one from Asia and one from South America.

**Table 1:** Patient characteristics and procedural details

Studies	Patients (*n*)	Male (%)	Mean age (years)	Hypertension (%)	Mean aneurysm diameter (mm)	Aneurysm type (*n*)	Mean procedure time ± SD (min)	Intraprocedural anticoagulation	Performed intervention	Treated vessels (*n*)	Procedural success
Fiorucci et al. [10]	61	66	70	78	69.7	TAAA (58) Suprarenal (3)	460 ± 147	ACT 250–350 s	B EVAR	NR	NR
Knowles et al. [18]	98	82	72	84	67.5	Juxtarenal (44) TAAA (27) Suprarenal (27)	327 ± 10	ACT > 300 s	F EVAR	457	NR
Xiao Hui et al. [19]	42	83	71	93	74	Juxtarenal (42)	184 ± 54	NR	Ch EVAR	56	100%
Wooster et al. [16]	50	83	77	90	NR	Paravisceral (50)	Perc. BA 183 ± 35, open BA 260 ± 122, AA 305 ± 71	ACT > 250 s	Ch EVAR	97	98%
Bosiers et al. [12]	425	64	76	89	NR	Juxtarenal (355) Pararenal (70)	220 ± 101	NR	Ch EVAR	723	NR
Coscas et al. [20]	16	88	73	69	62	Juxtarenal (16)	150 (120–360)	NR	Ch EVAR	26	94%
Ferreira et al. [21]	11	72	72	100	NR	TAAA (11)	3–8 h	NR	B EVAR	43	100%
Timaran et al. [22]	34	NR	NR	NR	NR	TAAA IV & Suprarenal (34)	NR	NR	F EVAR	126	NR
De Bruin et al. [23]	28	79	75	NR	66	Juxtarenal (20) Suprarenal (8)	185 (158–240)	5000 Units of heparin	Ch EVAS	47	NR
Lee et al. [24]	28	71	75	100	65	Juxtarenal (28)	202 (135–515)	ACT > 300 s	Ch EVAR	56	98%
Stanley et al. [28]	39	79	74	NR	59	Paravisceral (16) Juxtarenal (14) TAAA (9)	265 (168–348)	NR	F EVAR	102	NR
Mirza et al. [17]	243	74	75	NR	NR	TAAA (148) Pararenal (95)	NR	NR	F/B EVAR	838	99%
Ducasse et al. [25]	22	95	73	81	59	Juxtarenal (22)	105 (75–290)	5000 IU of heparin	Ch EVAR	NR	100%
Kotelis et al. [27]	39	92	74	87	62	TAAA IV (4) Suprarenal (12) Juxtarenal (23)	274 (193–626)	ACT > 250 s	F EVAR	106	95%
Ramanan et al. [26]	134	78	73	95	67	TAAA IV & Pararenal (74) TAAA I, II, III, V (60)	504	NR	B EVAR	491	NR
Gilling-Smith et al. [29]	6	66	71	NR	70*	TAAA (6)	600* (540–900)	5000 IU of heparin followed by personalized AC	F/B EVAR	23	NR

*AA* axillary artery, *AC* anticoagulation, *ACT* activated clotting time, *BA* brachial artery, *B EVAR* branched endovascular aortic repair, *Ch* chimney, *EVAS* endovascular aneurysm sealing, *F EVAR* fenestrated endovascular aortic repair, *F/B EVAR* fenestrated/branched endovascular aortic repair, *IU* international units, *NR* not reported, *perc.* percutaneous, *s* seconds, *TAAA* thoracoabdominal aortic aneurysm, * = median, @ technical success measured by cannulated side branches

Seven studies, which reported patients with left and right or bilateral upper extremity access, were included in the meta-analysis [11, 12, 18–20, 22, 24]. The sample size varied from 6 to 434 patients; included studies had a NOS score from 5 to 9 out of a maximal score of 9. The mean NOS score of the included studies was 6.5.

### Procedural Details

All studies stated which aortic intervention was performed. In total, 583 chimney EVAR [12, 16, 19, 20, 24, 25], 28 chimney endovascular aneurysm sealing (EVAS) [23], 210 fenestrated EVAR [18, 20, 22, 27, 28], 249 fenestrated/branched EVAR [11, 29] and 206 branched EVAR [10, 21, 26] were performed. The mean procedure time was reported by ten studies and ranged from 105 to 600 min. Eight studies presented information regarding their intraprocedural anticoagulation, ranging from the statement that heparinization took place to heparinization was performed until an activated clotting time between 250 and 350 s. For all types of included interventions, we pooled the mean intervention time. The mean intervention time for chimney EVARs was 214 min, for chimney EVAS 185 min, for fenestrated EVAR 298 min, for fenestrated/branched EVAR 600 min and for branched EVAR 489 min. In total, 2311 aortic side branches were included in the aneurysm repair. Four studies did not report how many aortic side branches were treated (Table 1) [10, 12, 25].

### Access Site

A total of 1276 patients underwent complex aortic interventions with left (*n* = 1028) [11, 12, 16, 18–29], right (*n* = 148) [10–12, 18, 22] or bilateral (*n* = 100) [12, 19, 20, 24] upper extremity access for the treatment of aortic aneurysms. One article had a discrepancy between the total number of patients and the size of each group, mentioning 425 treated patients but 434 used access strategies [12]. Totally, 114 patients were treated via the axillary artery and 709 via the brachial artery. In 27 patients, an axillary artery conduit was used. Two studies stated that they used both axillary and proximal brachial artery access, but did not differentiate the two access strategies in their results [12, 24]. A percutaneous approach was used in 59 patients in the brachial artery; the remaining 1317 access vessels were surgically exposed. The maximum induced sheath size was reported in ten studies. The sheath size ranged between 6Fr and 9Fr in 688 access vessels and between 10Fr and 12Fr in 386 access vessels (Table 2).

**Table 2:** Stroke rate and access complications

Study	N	Access vessel	Approach (*n*)	Sheath size upper access (*n*)	Access site			Cerebrovascular events			30-day Mortality (*n*)	Access complications
					*L*	*R*	BL	*L*	R	BL		
Fiorucci et al. [10]	61	BA	Open	10–12Fr		61			2		NR	Occlusion RA 1 Bleeding 3
Knowles et al. [18]	98	BA	Open (86) Perc. (12)	Mean 11Fr Median 12Fr	92	6		1	0		NR	Hematoma 4
Xiao Hui et al. [19]	42	BA	Perc.	6 or 7Fr	28		14	0		1	0	NR
Wooster et al. [16]	50	AA, BA	Perc. BA (5) Open BA (18) Conduit AA (27)	6Fr (24) 7Fr (21) 9Fr (1) 11Fr (3)	50			0			NR	AA Hematoma 1 (conduit). BA Hematoma 1, Neuropraxia 1
Bosiers et al. [12]	425	AA, BA	Open	6Fr (126) 7Fr (249) 8Fr (53)	308	49	77	4	1	2	23	NR
Coscas et al. [20]	16	BA	Open	7–10Fr	8		8	0		1	2	NC
Ferreira et al. [21]	11	AA	Open	12Fr	11			1			2	NC
Timaran et al. [22]	34	Prox. BA	Open	Mean 12Fr	17	17		0	1		0	NC
De Bruin et al. [23]	28	AA	Open	7Fr	28			3			1	NC
Lee et al. [24]	28	AA, BA	Open	7Fr	27		1	0		1	2	Neuropraxia 1
Stanley et al. [28]	39	BA	Not clearly reported	NR	39			0			0	NR
Mirza et al. [17]	243	Prox. BA, Distal BA, AA	Open	10–12Fr (159) 7–8Fr (84)	228	15		2	2		6	Dissection 5 Thrombosis 1 Hematoma 1 Transection 1 Neuropraxia 2
Ducasse et al. [25]	22	Prox. BA	Open	6Fr	22			1			1	NC
Kotelis et al. [27]	39	AA	Open	NR	39			2			3	NR
Ramanan et al. [26]	134	BA	Open	NR	134			3			5	NC
Gilling-Smith et al. [29]	6	BA	Open	NR	6			0			0	NR

*AA* axillary artery, *BA* brachial artery, *BL* bilateral, *Fr* French, *S* stroke, *L* left, *NC* no complications, *NR* not reported, *R* right, *RA* radial artery, *Perc.* percutaneous, *Prox.* proximal

### Results of the Included Studies

Technical success defined as successful side branch cannulation was reported by two studies and varied from 98 to 99% [11, 16]. Seven studies defined aneurysm exclusion as technical success and reported rates ranging from 94 to 100% [19–21, 24, 25, 27, 29]. Overall 51 patients (4.0%) died within 30 days after the performed intervention. Eleven studies reported upper extremity access site complications. Of all patients in these 11 studies, 22 (3%) access complications were reported consisting of three bleedings, five dissections, seven hematomas, four nerve injuries, one transection and two distal embolizations leading to radial artery occlusion.

All studies reported the number of cerebrovascular events their patients experienced; however, only five studies described how these were diagnosed. Three studies described that they reported only patients with symptoms of a cerebrovascular event and abnormalities on a brain CT scan [10, 12, 25]. Coscas et al. evaluated the cerebrovascular events by Rankin scale [30], and Knowles et al. stated that strokes were evaluated by a neurologist [18, 20]. The cerebrovascular complication rate ranged from 0 to 10.8%. Within the whole pooled population, a perioperative cerebrovascular complication rate of 2.2% was reported. Four cerebrovascular events were classified as TIA [12, 23]. Furthermore, four cerebrovascular accidents were classified as major and four as minor but without a statement about the recovery of the patient [10, 11, 20, 22]. Seven cerebrovascular accidents were reported to have fatal consequences [12, 19, 21, 22, 24]. Four studies did not make a statement about the severity of the event but reported that four patients with a cerebrovascular accident did fully recover, one patient recovered moderately and one patient did not recover at all [12, 23, 25, 27]. One article did not make a statement about the clinical severity or the recovery [26]. Four studies included in the review reporting cerebrovascular events did not mention the affected brain territory nor the patient’s clinical symptoms [11, 19, 22, 23]. Two studies reported cerebrovascular events in patients with left upper extremity access, which occurred in the right hemisphere [18, 27]. Fiorucci et al. [10] reported two strokes after the right upper extremity access of which one occurred in the left hemisphere. In all remaining cerebrovascular events, the access site matched the cerebral hemisphere of the cerebrovascular event.

The stroke rate for chimney EVAR was 1.9%, for chimney EVAS 10.7%, for fenestrated EVAR 1.9%, for fenestrated/branched EVAR 1.6% and for branched EVAR 2.9%. When comparing upper extremity access side, the cerebrovascular complication rate was 1.7% for patients treated via the left upper extremity access approach, 4% for a right-side approach and 5% for patients with a bilateral approach (Table 3).

**Table 3:** Stroke/TIA and type of aortic aneurysm

	Patients (*n*)	Stroke/TIA (%)	Stroke/TIA after left UEA (%)	Stroke/TIA after right UEA (%)	Stroke/TIA after bilateral UEA (%)
F EVAR	210	4 (1.9)	3 (1.5)	1 (4.3)	NP
B EVAR	206	6 (2.9)	4 (2.7)	2 (3.2)	NP
F/B EVAR	249	4 (1.6)	2 (0.8)	2 (13.3)	NP
Chimney EVAR	583	11 (1.9)	5 (1.3)	1 (2.0)	5 (5.0)
Chimney EVAS	28	3 (10.7)	3 (10.7)	NP	NP
Pooled	1276	28 (2.2)	17 (1.7)	6 (4.0)	5 (5.0)

*B EVAR* branched endovascular aortic repair, *EVAR* endovascular aortic repair, *EVAS* endovascular aortic sealing, *F EVAR* fenestrated endovascular aortic repair, *F/B EVAR* fenestrated/branched endovascular aortic repair, *NP* no patients, *TIA* transient ischemic attack, *UEA* upper extremity access

### Meta-Analysis

**Fig. 2:**
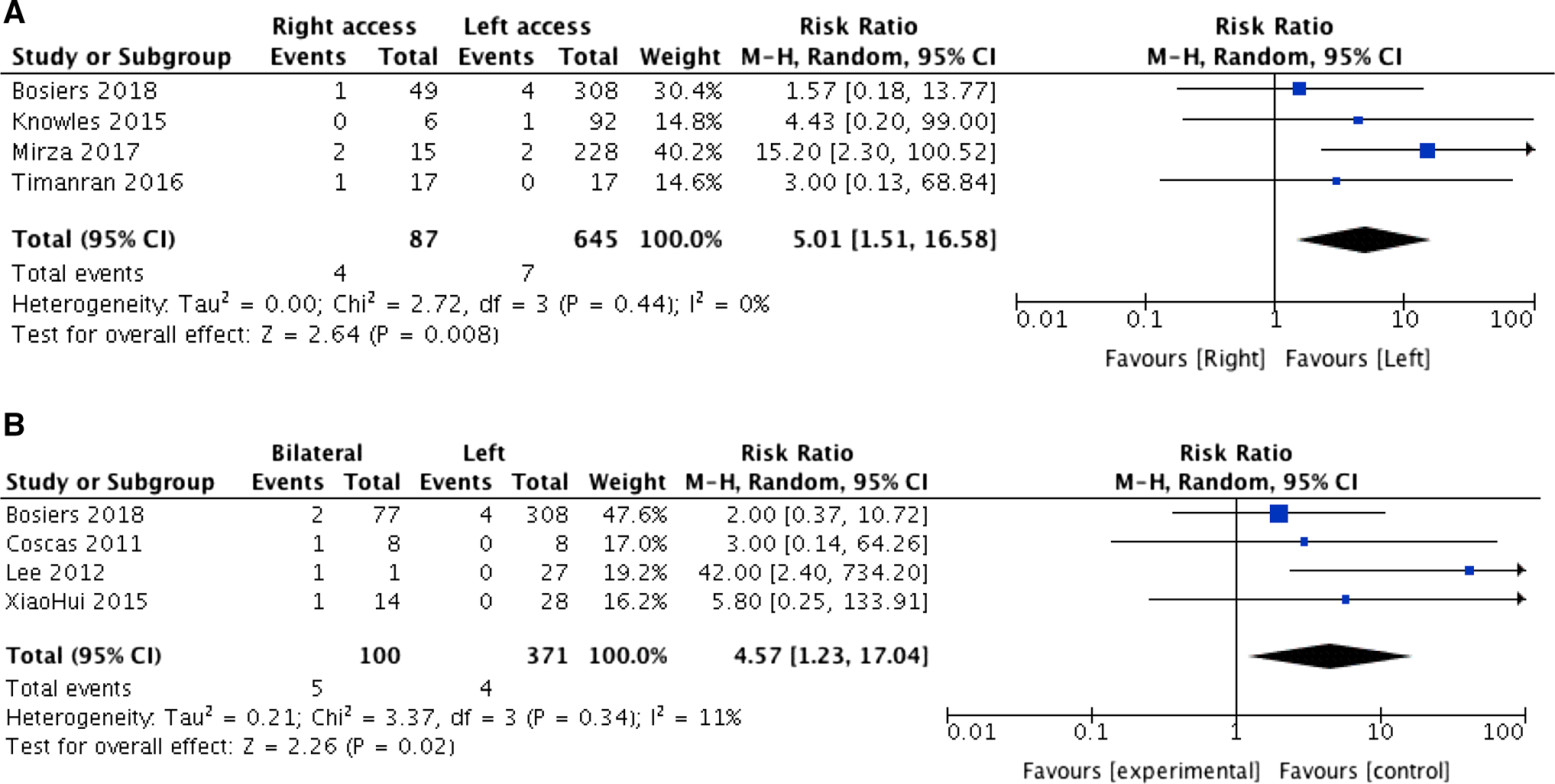
**A** Individual studies and pooled analysis showing a significant increase in the incidence of cerebrovascular events for complex EVARs partially performed through the right arm (*P* = 0.008). CI = confidence interval, HR = hazard ratio. **B** Individual studies and pooled analysis showing a significant increase in the incidence of cerebrovascular events for complex EVARs partially performed through bilateral access (*P* = 0.02). CI = confidence interval, HR = hazard ratio

Seven studies involving 645 patients treated from the left, 87 patients treated from the right and 100 patients treated through bilateral access were included in the meta-analysis. The pooled study populations were homogeneous for age, average amount of male patients and extent of aneurysm (Table 1). The risk to develop a perioperative cerebrovascular event was significantly increased in patients, who were approached via the right arm in comparison with the left-sided approached patients (RR 5.01, 95% CI 1.51–16.58, *P* = 0.008; Fig. 2A). The heterogeneity was low (*I*^2^ = 0%). Equally, the risk of a cerebrovascular event was significantly increased in patients with a bilateral approach (RR 4.57, 95% CI 1.23–17.04, *P* = 0.02; Fig. 2B) compared to left-sided approach, with again a low heterogeneity (*I*^2^ = 11%).

## Discussion

Stroke can be a devastating complication after complex endovascular treatment for aortic aneurysms, resulting in mortality, permanent disability and significant decline in quality of life. Although cerebrovascular events are multifactorial in etiology, upper extremity access is a recognized factor because catheter manipulations in the aortic arch and supra-aortic trunks can be associated with embolic events. Conversely, coronary intervention studies, comparing transradial and transfemoral access, were not able to identify access from the upper extremities as a risk factor for cerebrovascular events. Several experts advise approaching via the left upper extremity in complex aortic interventions to avoid crossing the aortic arch and supra-aortic trunks and potentially lower the risk of embolic events, but there is lack of clinical data to corroborate this hypothesis [31–33]. Moreover, a recent case series suggests that the right-sided upper extremity access is safe in properly selected patients undergoing complex aortic endovascular interventions [10]. We conducted a systematic review to evaluate the impact of upper extremity access laterality on the risk of cerebrovascular events and evaluated the influence of left, right or bilateral upper extremity access in patients undergoing complex aortic endovascular interventions in the recent literature.

It is known that complex aortic interventions have a higher stroke risk as compared to standard EVAR. The incidence of cerebrovascular events after EVAR is reported to be 0.6% according to Sharifpour et al. [34]. In a review from 2008, the stroke risk in thoracic EVAR ranged between 3.5 and 8.2% [9]. The highest risk of cerebrovascular events occurs with thoracic EVAR, especially when the repair extends into the aortic arch. Melissano et al. [35] reported a risk of stroke of 9.4%. For complex aortic repair involving incorporation of the renal–mesenteric arteries, the risk of stroke is estimated in 3.3% for branched EVAR, [10] 3.2% for chimney/snorkel EVAR [36–38] and 2.3% for fenestrated/branched EVAR 2.3% [39]. For the purpose of this review, we focused on patients who had these types of procedures and excluded aortic arch repair, because of the increased perceived risk of endovascular incorporation of the supra-aortic trunks.

In our systematic review, the perioperative cerebrovascular event rate ranged from 0 to 10.7% among the studies. Our meta-analysis showed that both right and bilateral upper extremity access significantly increased the risk of cerebrovascular events. Probably, manipulation through the aortic arch and thereby of the origins of both carotid arteries is responsible for the increased risk. Another suggested mechanism is hypoperfusion of the carotid and/or vertebral arteries due to partial occlusion by the sheath, especially in patients with small diameter or diseased vessels. Moreover, thrombus on the sheath can be dislodged into the cerebral circulation during sheath removal. Although we recognize that the risk of a cerebrovascular event may be affected by the diameter of the sheath, few or none of the reports provide this specific information. In general, large (12Fr) sheaths require access via the proximal brachial or axillary artery, whereas smaller sheaths (7–8Fr) are introduced in the distal brachial artery. Unfortunately, the included studies did not report their techniques and only some reported maximum sheath sizes, ranging from 5Fr to 12Fr. In previous reports, a correlation between the maximal sheath size and cerebrovascular event rate has not been found. Patient characteristics associated with a risk factor for cerebrovascular complications in complex EVAR are female sex, advanced age and paradoxically absence of a history of hypertension [40]. The studies we included in our review were comparable regarding gender distribution, age and history of hypertension. Buth et al. [40] and Bosiers et al. [12] found that a prolonged intervention time contributes to the risk to develop a stroke. Within the reviewed studies, the mean intervention time of fenestrated/branched EVAR was the longest. However, comparing the stroke rate in the fenestrated/branched EVAR studies to the other types of interventions, the stroke rates were almost similar.

The choice of the access site side is also influenced by a number of factors, including patient anatomy, location of the fixed imaging unit, ergonomics, radiation dose and physician preference. Not all patients are suitable for brachial access via right or left side. The type of aortic arch and presence of atherosclerotic debris within the ascending aorta, arch or supra-aortic trunks have direct effect on site selection. The location of a fixed or mobile imaging unit has direct influence on site selection and ergonomics. In most centers, the imaging gantry is positioned on the left side of the patient, whereas operators are often located on the right side of the patient. Therefore, it is more comfortable for operators to perform procedures via a right upper extremity access [5]. Another reason for the selection of right-side approach is radiation exposure, which is higher when the operator is located on the left side. Timaran et al. reported on 34 patients undergoing fenestrated EVAR for suprarenal or thoracic abdominal aortic aneurysms and found that a left upper extremity access led to a higher radiation exposure for the fellow and scrub nurse [22]. Left-sided radial artery access for coronary interventions has also been shown to lead to a higher radiation exposure for the operator [41], because radiation protective shields can hardly be placed correctly between the surgeon and the C-arm [10]. In addition, with a left-sided approach an additional scrub nurse is required as well as a tool table to hand over and store the catheters and wires, because the main femoral access is usually located on the right side.

In the literature, several other measurements besides a left-sided approach are described to increase the safety of upper extremity access. For every endovascular procedure, risk minimization starts with preoperative planning. Preoperatively, patients should be prescribed at least single antiplatelet therapy to reduce the cerebrovascular event risk. Unfortunately, the thrombocyte aggregation inhibition was underreported in the included studies. Furthermore, preoperative imaging of the aortic arch is indicated for the evaluation of arch calcification, atherosclerosis or mural thrombus [12]. During the intervention, it is advised to reduce the operation time and perform heparinization resulting in an activated clotting time above 250 s. Moreover, manipulation of wires and sheaths through the aortic arch should be minimized and a through-and-through wire can be used to stabilize the sheath while it is not used [10]. Three studies did not perform activated clotting time-based heparinization, two of those had a proportional high incidence of cerebrovascular events.

## Limitations and Quality of Evidence

The most relevant limitation of this study is the lack of a randomized comparison between right or left brachial access. Moreover, anatomical factors affecting the site selection were not specified in the studies, so it is likely that patients did not have comparable anatomy. A further limitation of this study is the small number of patients treated by right side or bilateral access. The studies included in the meta-analysis showed limited heterogeneity regarding the performed interventions, heparinization, aneurysm type, procedure time and induced sheath size. However, the patient population concerning age and gender distribution was homogenous. Another limitation is that most authors did not clarify how a cerebrovascular event was diagnosed, evaluated or treated. We did also include studies which were published at the end of the last or the beginning of this decade, and noted over time a decrease in complications after upper extremity access, probably due to the growing suitable inventory. Furthermore, it is unclear how experienced the interventionists were regarding upper extremity access of any kind. This might be important as there is a clear learning curve in upper extremity access, as it is known for the transradial access [42]. All of these factors influence the external validity of our findings.

## Conclusion

Right and bilateral upper extremity access increase the patients’ risk to develop a perioperative cerebrovascular event undergoing complex aortic aneurysm interventions compared to left upper extremity access.
